# The effects of exercise interventions on depressive symptoms in stroke patients: a systematic review and meta-analysis

**DOI:** 10.3389/fphys.2025.1492221

**Published:** 2025-03-17

**Authors:** Zhi-Yuan Wang, Ya-Lu Deng, Ting-Yuan Zhou, Zi-Yang Jiang, Yi Liu, Bo-Fu Liu, Yu Cao

**Affiliations:** ^1^ Department of Emergency Medicine and Laboratory of Emergency Medicine, West China Hospital, Sichuan University, Chengdu, Sichuan, China; ^2^ Institute for Disaster Management and Reconstruction, Sichuan University-The Hong Kong Polytechnic University, Chengdu, Sichuan, China

**Keywords:** exercise adherence, exercise duration, stroke, depression, meta-analysis

## Abstract

**Purpose:**

This systematic review and meta-analysis aimed to evaluate the effects of exercise interventions on depressive symptoms in stroke patients.

**Methods:**

Following PRISMA guidelines, We conducted searches in PubMed, Embase, CENTRAL, and Web of Science. The topic was the effect of exercise on depression levels in stroke patients. Patient’s performance on depression scales after exercise was assessed using standardized mean difference (SMD) and 95% confidence intervals (95% CI). A random effects model (RE) was used to conduct a meta-analysis and compare the results between subgroups conducted based on adherence to ACSM guidelines and the length of exercise interventions.

**Results:**

The analysis included 24 randomized controlled trials (RCTs) involving 1,757 participants. The meta-analysis revealed that exercise interventions had a significant positive effect on reducing depressive symptoms in stroke patients, with a standardized mean difference (SMD) of −0.43 (95% CI: 0.65, −0.21). Subgroup analysis indicated that high compliance with ACSM guidelines resulted in a more substantial reduction in depressive symptoms (SMD = −0.79, 95% CI: 1.10, −0.49), compared with low or uncertain compliance (SMD = −0.03, 95% CI: 0.16, 0.10). Longer exercise intervention was associated with greater mitigation of depressive symptoms. The differences between intervention groups of different lengths were statistically significant (p < 0.05).

**Conclusion:**

These findings support the integration of tailored exercise programs into post-stroke care to optimize mental health outcomes. Compliance to ACSM-recommended exercise dosages significantly ameliorate depression levels in stroke patients. Further research is warranted to explore standardized exercise regimens in larger, multicenter trials.

**Systematic Review Registration:**

https://www.crd.york.ac.uk/prospero/#recordDetails, identifier PROSPERO(CRD42024579095).

## 1 Introduction

Depression is a prevalent and debilitating condition that affects approximately one-third of all stroke survivors (Hackett and Pickles, 2014; Medeiros et al., 2020), significantly impairing their quality of life and rehabilitation. Based on a complex interplay of psychological, biological, and social factors, a spectrum of depressive symptoms persisting long after stroke onset (Das and Rajanikant, 2018; Hadidi et al., 2009). The etiology of depression involves brain lesions, neurochemical alterations, inflammation, and the psychological impact of the stroke itself (Medeiros et al., 2020). Brain lesions from stroke can damage areas responsible for emotion regulation (Douven et al., 2017; Liang et al., 2018), while neurochemical alterations disrupt neurotransmitters like serotonin and dopamine (Berretta et al., 2014; Levy et al., 2018), which are crucial for mood regulation. Inflammation following a stroke can interfere with neurogenesis, further exacerbating depressive symptoms (Loubinoux et al., 2012; Marx et al., 2023), and the psychological impact of losing independence and dealing with ongoing health issues contributes to feelings of hopelessness and isolation (Woodford et al., 2018; Narendrula et al., 2023). Despite its high incidence rate and adverse effects to daily life, it still remains inadequately treated in most clinical settings (Skolarus et al., 2017; El Husseini et al., 2012; Paul et al., 2006), which warrants effective interventions to be seamlessly integrated into post-stroke care.

Exercise has been recognized as a promising non-pharmacological intervention for managing depression (Lai et al., 2006; Lanctôt et al., 2020). The therapeutic effects of exercise on mental health are well-documented (Immink et al., 2014; Koch et al., 2020; Taylor-Piliae et al., 2014), with numerous studies demonstrating its efficacy in reducing depressive symptoms through mechanisms such as regulating neurotransmitters (Okaty et al., 2019; Meneses, 1998; Tang et al., 2022), reducing inflammation (Tang et al., 2022; Niu et al., 2020), enhancing neuroplasticity, and improving overall physical health (Sims et al., 2009). Given the chronic nature of depression and the potential side effects of pharmacological treatments, exercise presents a viable and sustainable alternative or adjunct therapy for patients with depression (Heissel et al., 2023; Herring and Meyer, 2024). The American College of Sports Medicine (ACSM) provides comprehensive guidelines on exercise prescription, encompassing frequency, intensity, type, and duration to optimize health outcomes (Garber et al., 2011). Recommendations include aerobic exercise 3–5 days a week at moderate to vigorous intensity, and resistance training 2–3 days per week. Aerobic activities such as walking or cycling should be done for 150 min of moderate or 75 min of vigorous activity per week. Resistance training sessions should last 20–60 min. These guidelines aim to enhance both physical and mental wellbeing, offering a sustainable, alternative or adjunct therapy to pharmacological treatments for depression in healthy people (Rosenbaum et al., 2014; Aaron et al., 2016). However, these guidelines have not been applied to stroke survivors. Exercise helps stroke patients improve physical strength and daily functioning, boosting self-confidence (Pontes et al., 2019). It also promotes the production of brain-derived neurotrophic factor, cortisol, and β-endorphins, alleviating depressive symptoms (Fichna et al., 2007; Foley and Fleshner, 2008; Li et al., 2024). These findings support the role of exercise in improving depression symptoms in stroke survivors, but the specific effectiveness of exercise in alleviating depression in this population remains unclear.

Several meta-analyses have investigated the effects of exercise on depression in the general population (Pearce et al., 2022; Noetel et al., 2024). These meta-analyses found that physical activity, including walking, yoga, and resistance training, significantly reduces depression risk and symptoms. Increasing exercise duration and intensity further enhances these benefits, with exercise being an effective alternative or adjunct treatment for depression. A network meta-analysis of non-pharmacological treatments for post-stroke depression (PSD) showed that, among the 33 included therapies, exercise therapy, while not the most effective treatment, was still effective in improving post-stroke depression symptoms (Yi et al., 2024). Previous meta-analyses on the impact of exercise in stroke patients with depression have shown that both Tai Chi and aerobic exercise are effective in alleviating depressive symptoms after stroke (Chen et al., 2024). Structured exercise interventions have been found to significantly reduce depressive symptoms during subacute and chronic stages of stroke and improve short-to medium-term quality of life (Eng and Reime, 2014; Song et al., 2023). However, the relationship between the frequency, intensity, and duration of exercise and the improvement depression remains inconclusive. Therefore, understanding the dose-response relationship between exercise and depression in stroke patients is crucial for maximizing treatment effectiveness while minimizing the risks of overtraining or injury (Tseng et al., 2007). This systematic review and meta-analysis aims to address this gap by evaluating the effects of exercise interventions, based on ACSM guidelines, in stroke patients. By systematically reviewing and analyzing the available literature, this study aims to evaluate the effects of exercise interventions on depressive symptoms in stroke patients. The findings from this review will contribute to the development of evidence-based exercise prescriptions for implementation in clinical practice, thereby improving the mental health and overall wellbeing of stroke survivors.

## 2 Methods

This review strictly adhered to the guidelines outlined in the Preferred Reporting Items for Systematic Reviews and Meta-Analyses (PRISMA) (Page et al., 2021) and was registered in PROSPERO (CRD42024579095).

### 2.1 Search strategy

A comprehensive search included the following four electronic databases: PubMed (since 1996), Embase (since 1947), CENTRAL (since 1999), and Web of Science (since 1960), with the search period ranging from the inception of each database to 9 June 2024. The search strategy was carefully designed based on the PICOS principle. (P) Population: Patients (≥18 years) with a definite diagnosis of ischemic or hemorrhagic stroke, including acute, subacute, or chronic stages after stroke. (I) Interventions: Invention group should receive a full range of land exercise therapy. (C) Comparison: Control group received usual care, educational guidance, rehabilitation training, mild activity or no treatment. (O) Outcomes: Patients’ level of depression after intervention. (S) Study design: Randomized controlled trial (RCT). The search included a combination of MeSH terms and free words. We also searched the reference lists of included studies and relevant reviews to identify additional potential studies. If necessary, we will contact the author for further information. Supplementary Table provides all of detailed overview of the search strategy.

### 2.2 Inclusion and exclusion criteria

Inclusion criteria: (1) Study subjects (aged 18 years and above) with a definitive diagnosis of ischemic or hemorrhagic stroke. Mixed studies were also included when data on patients who suffered from a stroke could be extracted; (2) The experimental group completed a series of land exercises (Guo et al., 2022), including strength training, flexibility training, aerobic training, and a mixture of multiple exercises; (3) The control group received usual care, educational guidance, rehabilitation training, mild activity or no treatment; (4) Outcome focused on the first results of depression test after intervention; and (5) RCTs.

Exclusion criteria: (1) Subjects have severe aphasia, severe vision or hearing impairment; (2) Study subjects cannot complete the depression test due to severe intellectual disability or severe comorbidities; (3) baseline characteristics of patients in control group are significantly different from those in intervention group; (4) Animal research; (5) Reports, protocols, reviews, case reports, and conference papers, among others; (6) Failure to obtain the full text; (7) Duplicate publications; (8) Data that could not be extracted.

### 2.3 Study selection

The use of Endnote software streamlined the process of screening and managing the vast array of literature available. Initially, two authors, Z.W. and Y.D., conducted an independent screening of titles and abstracts. Meanwhile, they excluded any duplicated entries as well as literature types such as reviews, conference abstracts, correspondence, case reports, protocols, animal studies, and non-randomized controlled trials (non-RCTs). This initial filtering ensured that only relevant studies progressed to the next step. Then, both researchers undertook a thorough re-evaluation of the remaining abstracts to apply the aforementioned inclusion and exclusion criteria. After assessment, the two researchers conducted an review of all included articles. They analyzed the content of each study to decide its suitability for publication. In instances where there was any disagreement or inconsistency between the two researchers, they consulted with the third author, T.Z., facilitating a discussion that led to a consensus and a final decision. Notably, this entire process was conducted without any restrictions regarding the release date or language of the literature, ensuring a comprehensive and inclusive review.

### 2.4 Data extraction

Two researchers, Z.W. and Y.D., carried out a comprehensive and independent extraction of relevant data utilizing a standardized pre-designed form. The data collection encompassed publication characteristics (title, first author, published year, and country of origin), group design, sample size, and specific intervention. importantly, the details of the exercise interventions including details of the type, duration, frequency, intensity levels, repetition rates and overall time were accurately documented.

In addition, demographics, outcome of all subjects were extracted. Following data extraction, both raters, Z.J. and Y.L., independently assessed exercise dose—factoring in frequency, intensity, workload, duration, and other relevant components—as well as the adherence to exercise interventions specifically in patients, in accordance with the guidelines set forth by the American College of Sports Medicine (ACSM) (Garber et al., 2011) (Table 1). The scoring criteria for each indicator in this meta-analysis was 0 points for not completely meeting the standards; 1 point for not being sure whether it meets or may meet the standards; 2 points for completely meeting the standards. Utilizing this grading system, the researchers calculated the adherence level to the ACSM guidelines regarding exercise dosing in each individual study. A compliance rate of 70% or higher was regarded as representative of high adherence to ACSM recommendations (Wen et al., 2024; Niu et al., 2024), while a rate falling below this threshold was interpreted as indicative of either low or uncertain compliance. In instances where there were discrepancies in ratings or assessments, a third reviewer, B.L., joined the discussion to facilitate a consensus and ensure the accuracy of the evaluations made.

**TABLE 1:** The ACSM recommendations for cardiorespiratory fitness, muscular strength and flexibility in apparently healthy adults.

Exercise dose	Cardiorespiratory exercise	Resistance exercise	Flexibility exercise
Frequency	3–5 days per week	2–3 days per week	≥2–3 days per week, daily
Intensity/ workload	40%–60% VO_2_R or HRR; RPE of 12–13 on a 6–20 scale	Start with 40%–50% 1RM, more capable with 60%–70% 1RM	Stretch until you feel your muscles being pulled tight or a slight discomfort
Duration	Continuous or cumulative 30 min	≥1 group, 8–12 repetitions	Keep static pulling for 10–30 s; repeat 2–4 times

Note: VO_2_R, oxygen uptake reserve. HRR, heart rate reserve. RPE: ratings of perceived exertion.

### 2.5 Risk of bias of individual studies

Two raters (T.Z. and B.L.) independently assessed the risk of bias (ROB) using the Cochrane Bias Risk Assessment Tool for RCTs. Seven domains were considered: (1) randomized sequence generation, (2) allocation concealment, (3) blinding of participants and personnel, (4) blinding of outcome assessment, (5) incomplete outcome data, (6) selective reporting, and (7) other bias. Trials were categorized into three levels of ROB by the number of components for which high ROB potentially existed: high risk (five or more), moderate risk (three or four) and low risk (two or less) (Higgins et al., 2011).

### 2.6 Data analysis

Meta-analyses were conducted using STATA 15.1 for comparing the results of the included studies. In included studies, all variables were continuous and reported as means with standard deviation (SD).

On the one hand, we divided studies into three subgroups according to the length of intervention (Length < 1-month, 1-month ≤ Length < 3-month, Length ≥ 3-month). On the other hand, subgroup analyses of high and low or uncertain compliance groups were performed. The heterogeneity among studies within each subgroup was assessed using the Higgins I^2^ statistic, as suggested by the Cochrane Handbook (Higgins et al., 2024). The level of heterogeneity was categorized as low (0 < I^2^ ≤ 30%), moderate (30% < I^2^ ≤ 60%), or high (I^2^ > 60%). Due to the differences in the types of interventions included in this study, such as variations in exercise frequency, duration, and the diverse methods used for outcome assessment, we applied a random-effects model in the statistical analysis, considering the clinical and methodological heterogeneity. This was represented by the standardized mean difference (SMD) along with a 95% confidence interval (95% CI). If heterogeneity is high, we will further perform meta-regression analysis to explore the source of heterogeneity by examining potential study characteristics that may cause heterogeneity. The possibility of publication bias was assessed by constructing funnel plots of the effect sizes of each study relative to the standard error, and asymmetry was tested using Begg’s rank correlation test and Egger’s linear regression test, with *p* < 0.05 indicating statistical significance. Sensitivity analyzes were also performed to test the robustness of the findings by excluding each study one by one.

## 3 Results

### 3.1 Study selection/literature search

**FIGURE 1:**
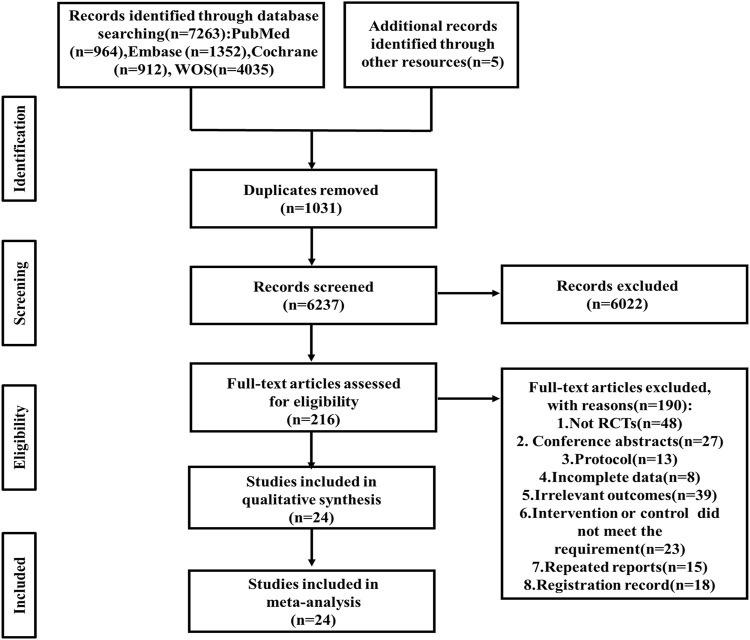
Relevant articles included in this review. WOS, Web of science; RCT, randomized controlled trial.

A total of 7,263 articles were retrieved from four databases (PubMed 964, Embase 1,352, Cochrane Library 912, Web of Science 4,035), with an additional 5 documents manually sourced from other resources. After removing duplicates, 6,237 records met the inclusion criteria. Subsequently, following a thorough review of the titles and abstracts, 216 remaining articles were fully evaluated. 48 Non-RCT studies, 27 conference abstracts, 13 protocols, 8 studies with incomplete data, 39 studies involving irrelevant outcomes, 23 studies with undesired intervention or control, 15 repeated reports and 18 registration records were excluded. Finally, after a comprehensive examination, 24 relevant articles were included in this review (Lai et al., 2006; Immink et al., 2014; Koch et al., 2020; Taylor-Piliae et al., 2014; Sims et al., 2009; Mead et al., 2007; Steen Krawcyk et al., 2019; Aguiar et al., 2020; Aidar et al., 2014; Chaiyawat and Kulkantrakorn, 2012; Cumming et al., 2008; Deijle et al., 2022; Faulkner et al., 2015; Holmgren et al., 2010; Hong, 2011; Jun et al., 2013; Liu et al., 2024; Smith and Thompson, 2008; Sun et al., 2022; Van De Port et al., 2012; Vloothuis et al., 2019; Zhao et al., 2020; Mishchenko and Mishchenko, 2022; Zhang et al., 2018) (Figure 1).

### 3.2 Study characteristics

A total of 24 studies encompassing 1,757 patients were included. they had an average age of 63 years, and 42% were female. The intervention group consisted of 906 participants, while the control group included 851 participants. The smallest sample size included in the studies was 20 (Smith and Thompson, 2008), and the largest was 250 (Van De Port et al., 2012). Except for four studies that included patients diagnosed with post-stroke depression (Zhang et al., 2018; Sun et al., 2022; Sims et al., 2009; Liu et al., 2024), the remaining studies focused on patients in the recovery phase of either acute or chronic stroke.

In all studies, the duration of exercise interventions ranged from 2 weeks to 12 months, with the minimum frequency of once a month (Chaiyawat and Kulkantrakorn, 2012) and the maximum of twice daily (Cumming et al., 2008). The types of interventions mainly included aerobic exercise, resistance training, balance training, vibration exercise, Tai Chi, Baduanjin, and yoga. After categorizing the interventions, 14 studies involved the exercise dosage of cardiorespiratory exercise, 7 studies involved the exercise dosage of resistance training, 13 studies involved the exercise dosage of flexibility training, and 8 studies involved two or more exercise modalities (Aidar et al., 2014; Deijle et al., 2022; Faulkner et al., 2015; Koch et al., 2020; Lai et al., 2006; Mead et al., 2007; Sims et al., 2009; Vloothuis et al., 2019)

In the section of outcomes, data were extracted from the immediate post-intervention evaluation despite studies with multiple follow-up assessments. The three most commonly used questionnaires were the Hospital Anxiety and Depression Scale (HADS), the Centre for Epidemiologic Studies Depression Scale (CES-D), and the Geriatric Depression Scale (GDS), which were used 6, 5, and 4 times, respectively (Table 2).

**TABLE 2:** Characteristics of the studies included in the meta-analysis.

Author	Country	Year	Population	Age Mean (SD)	Total/male/female	Intervention	Control	Outcome
Smith	United States	2008	Ischaemic stroke more than 3 months but less than 2 years	T:57.8 (7.0) C:56.0 (8.3)	T:10/8/2 C:10/4/6	Treadmill training Length: 4 weeks Frequence: 3 times a week Duration: 20-min	Educational guidance	BDI
Sims	Australia	2008	Depressed chronic stroke survivors more than 6 months	T:67.95 (14.76) C:66.27 (16.01)	T: 23/14/9 C: 22/13/9	Progressive resistance training Length: 10 weeks Frequence: 2 times a week Duration:	Usual care	CES-D
Sun	China	2022	PSD diagnosed between the first and 18th month after stroke	T: 62.03 (7.37) C: 65.23 (6.29)	T: 30/17/13 C: 30/17/13	Yijinjing Qigong exercise Length: 3 weeks Frequence: once a day Duration: 60-min	Rehabilitation training	HAMD-24
Lai	United States	2006	Stroke survivors who had completed acute rehabilitation	T: 68.5 (9.0) C: 70.4 (11.3)	T: 44/23/21 C: 49/27/22	Progressive, structured, physical exercise Length: 12 weeks Frequence: 3 times a week Duration: 90-min	Usual care	GDS
Holmgren	Sweden	2010	Consecutive,≥55 years patients with risk of falls at 3–6 months after stroke	T: 77.7 (7.6) C:79.2 (7.5)	T: 15/9/6 C: 19/12/7	High-intensive exercise Length: 5 weeks Frequence: 3 times a week Duration: 90-min	Educational guidance	GDS
Aidar	Portugal	2014	Stroke over a year	T: 51.7 (8.0) C: 52.5 (7.7)	T: 11/6/5 C: 13/9/4	Strength training Length: 12 weeks Frequence: 3 times a week Duration: 60-min	No exercise	BDI
Hong	Korea	2011	Stroke survivors more than 6 months	T: 57.2 (8.5) C: 57.0 (9.7)	T: 20/9/11 C: 20/13/7	Upper extremity exercise Length: 6 weeks Frequence: 3 times a week Duration: 50–60 min	No exercise	CES-D
Zhang	China	2018	PSD conformed both Western and Chinese medicine’s diagnostic criteria	T: 63.7 (6.8) C: 62.4 (7.6)	T: 45/27/18 C: 45/25/20	Tai Ji Quan plus acupuncture Length: 48 weeks Frequence: 5 times a week Duration: 40-min	No exercise	HAMD-24
Liu	China	2024	Stroke was 2 weeks–6 months; Conforming to PSD	T: 58.86 (10.83) C: 56.22 (11.54)	T: 50/31/19 C: 50/29/21	Baduanjin with psychotherapy Length: 8 weeks Frequence: twice a day Duration: 30-min	No exercise	HAMD-24
Koch	United States	2020	Stroke within 1 year	T: 59 (11) C: 58 (12)	T: 86/60/26 C: 45/21/24	Combined aerobic, resistance, and cognitive training Length: 12 weeks Frequence: 3 times a week Duration: 40–60 min	Mild activity	CES-D
Jun	Korea	2013	Acute ischaemic stroke hospitalised less than 2 weeks	T: 60.70 (8.59) C: 55.10 (17.23)	T: 15/6/9 C: 15/9/6	Music-movement therapy Length: 8 weeks Frequence: 3 times a week Duration: 60-min	Usual care	CES-D
Mishchenko	Ukraine	2022	3–6 months from the moment of a vascular accident	T: 65 (6.67) C: 66 (6.67)	T: 43/23/20 C: 41/21/20	Physical rehabilitation Length: 3 weeks Frequence: 5 times a week Duration: 3-h	No exercise	BDI
Chaiyawat	Thailand	2012	3 days after stroke onset	T: 67 (10) C: 66 (11)	T: 30/14/16 C: 30/13/17	Home rehabilitation Length: 6 months Frequence: once a month Duration: 6–15 min	Rehabilitation training	HADS
Zhao	China	2022	First-ever stroke, in the subacute stage of stroke	T: 62.61 (12.88) C: 63.35 (12.90)	T: 80/42/38 C: 80/39/41	Sitting Tai Chi Length: 12 weeks Frequence: 3 times a week Duration: 40-min	Mild activity	GDS
Vloothuis	Netherlands	2019	Stroke patients were planned to be discharged	T: 60.53 (14.82) C: 59.26 (15.01)	T: 32/21/11 C: 34/20/14	Caregiver-mediated exercises Length: 8 weeks Frequence: 5 times a week Duration: 60-min	Usual care	HADS
van de Port	Netherlands	2012	Stroke patients were discharged home and need to continue physiotherapy	T: 56 (10) C: 58 (10)	T: 126/82/44 C: 124/80/44	Circuit training Length: 12 weeks Frequence: 2 times a week Duration: 90-min	Usual care	HADS
Taylor-Piliae	United States	2014	Survivors were at least 3 months poststroke	T: 71.5 (10.3) C: 68.2 (10.3)	T: 53/34/19 C: 48/23/25	Tai Chi training or strength and range of movement Length: 12 weeks Frequence: 3 times a week Duration: 60-min	Usual care	CES-D
Aguiar	Brazil	2020	Stroke>6 months	T: 52 (11) C: 48 (10)	T: 11/8/3 C: 11/8/3	Aerobic treadmill training (60–80%HRR) Length: 12 weeks Frequence: 3 times a week Duration: >30-min	Mild activity	PHQ
Krawcyk	Denmark	2019	Within 21 days after onset of stroke symptoms	T: 63.7 (8.9) C: 63.7 (9.2)	T: 31/23/8 C: 32/26/6	High-intensity interval training Length: 12 weeks Frequence: everyday Duration: >25-min	Usual care	MDI
Mead	UK	2007	Patients had completed usual rehabilitation	T: 72.0 (10.4) C: 71.7 (9.6)	T: 32/18/14 C: 34/18/16	Progressive endurance and resistance training Length: 12 weeks Frequence: 3 times a week Duration: 15–40 min	Mild activity	HADS
Immink	Australia	2014	Stroke>9 months and resulting in hemiparesis	T: 56.1 (13.6) C: 63.2 (17.4)	T: 11/6/5 C: 11/3/8	Yoga training Length: 10 weeks Frequence: 1 time a week Duration: 90-min	No exercise	GDS
Faulkner	New Zealand	2015	TIA or mild/non-disabling within 7 days of stroke onset	T: 65 (11) C: 68 (10)	T: 27/15/12 C: 28/14/14	Early exercise Length: 8 weeks Frequence: 2 times a week Duration: 90-min	Usual care	HADS
Deijle	Netherlands	2022	TIA or minor ischemic stroke less than 1 month	T: 64.7 (8.9) C: 63.9 (10.6)	T: 60/34/26 C: 59/36/23	Aerobic and strength training Length: 12 weeks and 9-month follow-up Frequence: 2 times a week Duration: 60-min	Usual care	HADS
Cumming	Australia	2008	Patients were admitted within 24 h of symptom onset	T: 74.6 (14.6) C: 74.9 (9.8)	T: 38/22/16 C: 33/16/17	Very early mobilization Length: 2 weeks Frequence: 2 times a day Duration: NA	Usual care	IDA

### 3.3 Risk of bias

**FIGURE 2:**
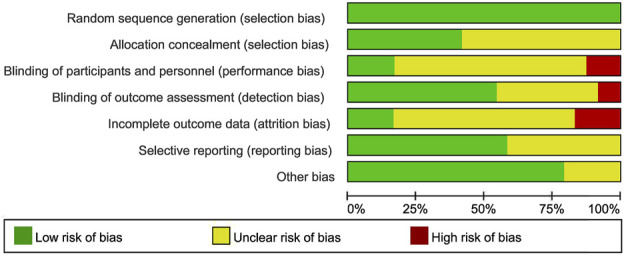
Combined percentage risk of bias in each risk domain for all included trials.

**FIGURE 3:**
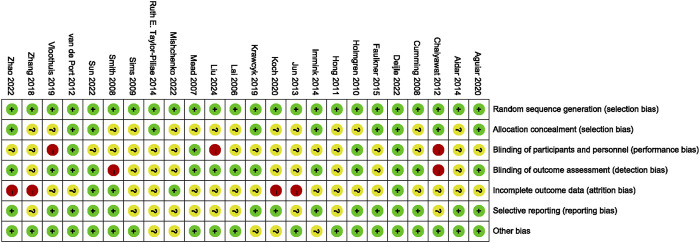
Risk of bias summaries for all exercise trials.

All studies were considered to have a low risk of bias in generating random sequences. 10 studies explicitly described allocation concealment and were therefore judged to have a low risk of bias. The risk of blinding bias was relatively high. Three studies were regarded as a high risk of bias due to the inherent difficulty of implementing double-blind conditions in exercise interventions (Chaiyawat and Kulkantrakorn, 2012; Liu et al., 2024; Vloothuis et al., 2019). 13 studies clearly defined the blinding of outcome assessors had a low risk of bias. In terms of incomplete outcome data, 20 studies reported differences in participant numbers between baseline and post-intervention assessments. Five studies exhibited significant differences in participant numbers pre- and post-intervention (≥10 participants) had a high risk of bias. However, selective reporting bias posed the unclear risk in 10 studies, as they either did not report their pre-registered plans or failed to provide detailed explanations for participant withdrawals. Additionally, 5 studies might have other risks of bias (Figures 2, 3).

### 3.4 Compliance with the ACSM recommendations

Based on the level of compliance, studies were categorized into two groups (Wen et al., 2024; Niu et al., 2024): high compliance and low or uncertain compliance. In 12 studies, the exercise interventions met the ACSM recommendations, with adherence rates of 70% or higher. Conversely, in 12 studies, the adherence rate was below 70% (Table 3). Additionally, the lack of sufficient information on exercise prescription hindered the ability to conduct a thorough assessment.

**TABLE 3:** Exercise interventions evaluated according to the American College of Sports Medicine’s (ACSM) recommendations.

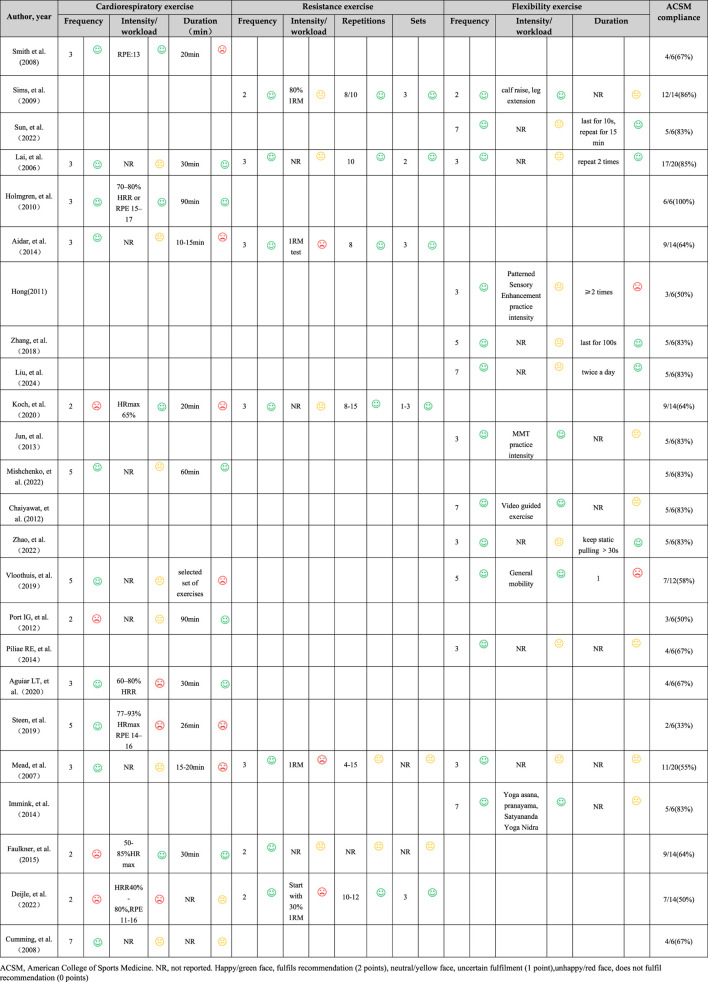

### 3.5 Meta-analysis

**FIGURE 4:**
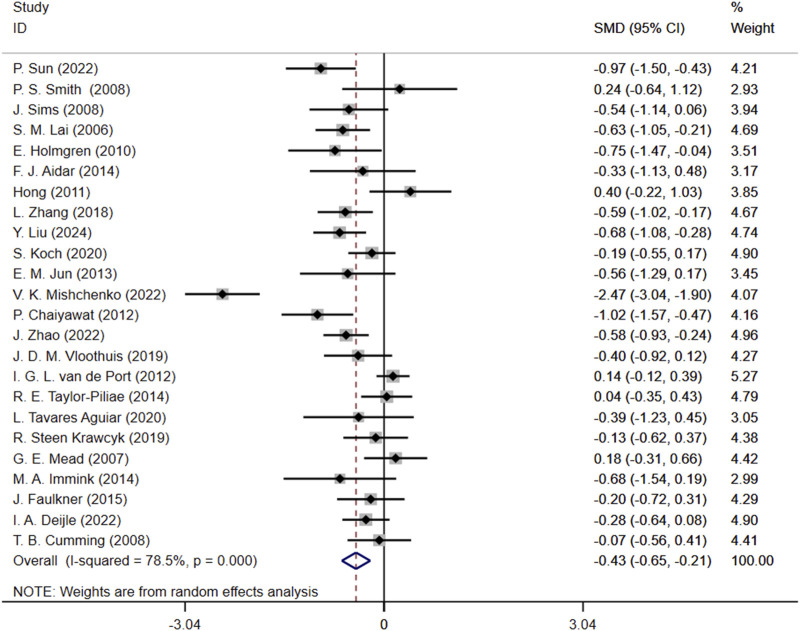
Forest plot of the effect of exercise on depression in patients SMD, Standardized mean difference; CI, Confidence intervals.

Initially, we conducted an overall heterogeneity test on the included studies and found a high level of heterogeneity among the studies (I^2^ = 78.5%, p = 0.000). Consequently, a random-effects model was employed for the statistical analysis. The data analysis revealed an overall standardized mean difference (SMD) of −0.43 (95% CI: 0.65, −0.21), indicating that exercise interventions are beneficial in alleviating depression symptom for stroke patients (Figure 4).

**FIGURE 5:**
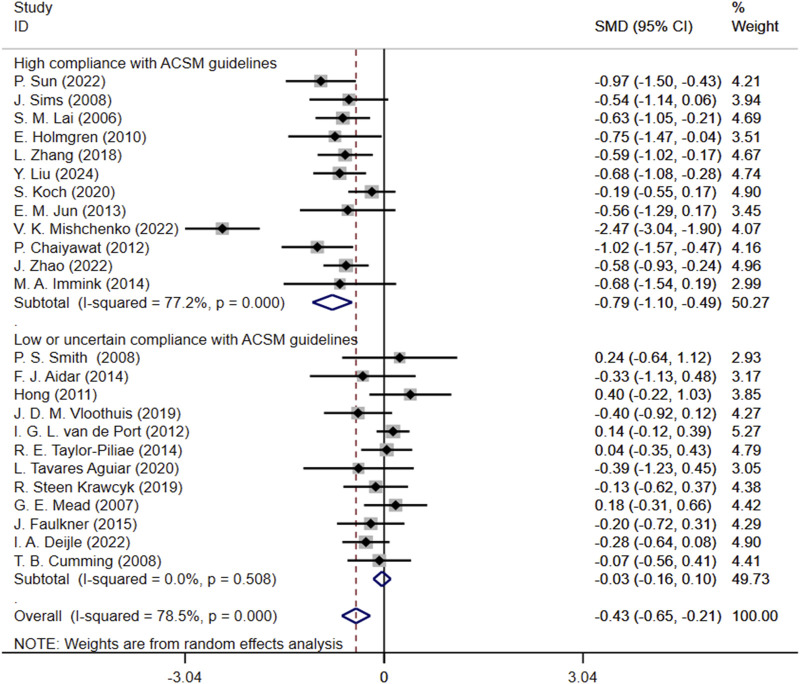
Forest plot for subgroup analysis based on ACSM guideline compliance. MD, Standardized mean difference; CI, Confidence intervals; ACSM, American College of Sports Medicine.

Subgroup analysis showed that SMD for high compliance subgroup was −0.79 (95% CI: 1.10, −0.49), while SMD for low or uncertain compliance subgroup was −0.03 (95% CI: 0.16, 0.10). These results suggest that exercise interventions with high compliance to ACSM guidelines may alleviate depression. In contrast, the confidence interval for the low or uncertain compliance subgroup crossed zero, indicating that it is unclear whether these interventions have a significant effect on improving depression. The heterogeneity among studies was 77.2% in the high compliance subgroup, whereas it was 0% in the low or uncertain compliance subgroup (Figure 5).

**FIGURE 6:**
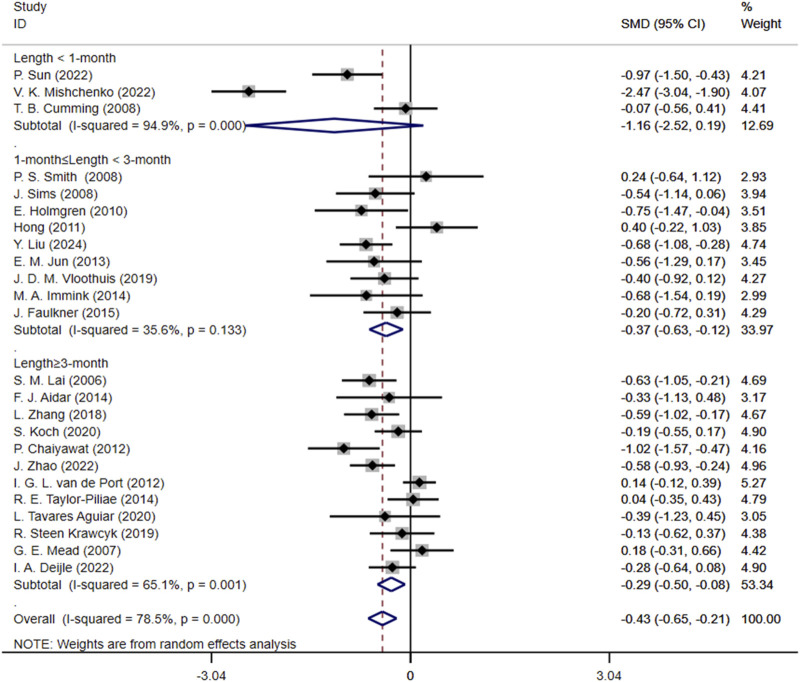
Forest plot for subgroup analysis based on exercise length. SMD, Standardized mean difference; CI, Confidence intervals.

Another subgroup analysis based on the length of interventions showed that for Length<1-month group, SMD was −1.16 (95% CI: 2.52, 0.19), with a high heterogeneity of 94.9%; for 1-month ≤ Length<3-month group, SMD was −0.37 (95% CI: 0.63, −0.12), with a heterogeneity of 35.6%; for Length≥3-month group, SMD was −0.29 (95% CI: 0.50, −0.08), with a heterogeneity of 65.1% (Figure 6).

**FIGURE 7:**
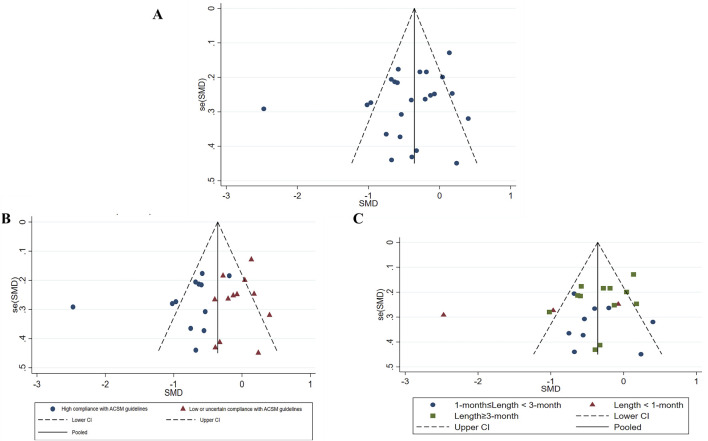
Study’s Funnel plot of publication bias **(A)**, Funnel plot of all studies **(B)**, Publication bias of subgroup analysis (based on ACSM guideline compliance) **(C)**, Publication bias in subgroup analysis (based on exercise length). SMD, Standardized mean difference; CI, Confidence intervals; ACSM, American College of Sports Medicine.

**FIGURE 8:**
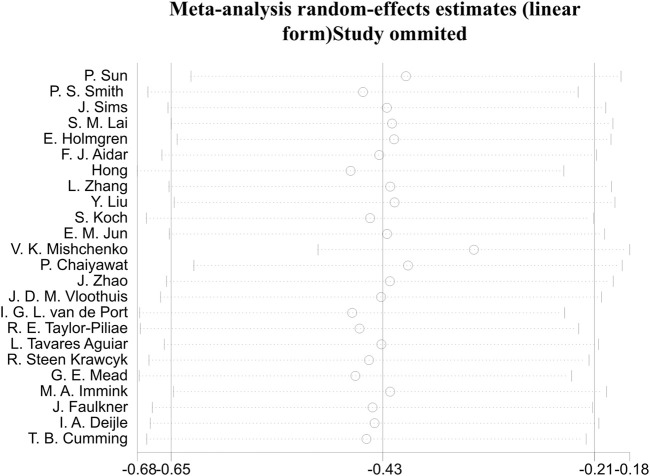
Sensitivity analysis of included studies.

In assessment of publication bias and sensitivity analysis, the funnel plot appeared to be roughly symmetrical, indicating no significant evidence of publication bias (Figure 7). This finding was further supported by the Begg’s test (*p* = 0.568) and Egger’s test (*p* = 0.164), both of which provided additional evidence against the presence of substantial publication bias. Sensitivity analysis demonstrated that no single study had a significant impact on the overall results, thereby confirming the robustness of our findings (Figure 8). The regression analysis results indicated that, after adjusting for covariates including sample size (*p* = 0.322), participant gender (*p* = 0.292), age (*p* = 0.657), type of scale (*p* = 0.243), country (*p* = 0.681), and overall _cons (*p* = 0.903), we did not find any significant improvement in depression among stroke patients attributable to the exercise interventions (Table 4).

**TABLE 4:** Univariate meta-regression analysis of the impact of different study characteristics on between-study heterogeneity.

Outcome	Covariates	Regression coefficient	Standard error	t	P > |t|	[95% Cof. Intervl]
Depression Level	Sample size	−0.0183163	0.0184924	−0.99	0.322	[-0.0545608, 0.0179282]
	Sex	0.0295511	0.0280613	1.05	0.292	[-0.0254481, 0.0845502]
	Age	−0.0089493	0.0201538	−0.44	0.657	[-0.04845, 0.0305514]
	Scale type	0.1071921	0.0917473	1.17	0.243	[-0.0726293, 0.2870135]
	Country	−0.0156045	0.03802	−0.41	0.681	[-0.0901224, 0.0589134]
	_cons	−0.1413714	1.162174	−0.12	0.903	[-2.419191, 2.136448]

## 4 Discussion

This systematic review with meta-analysis evaluated the effects of exercise interventions on depressive symptoms in stroke patients based on ACSM guidence. Our study supports the positive impact of exercise interventions on alleviating post-stroke depression. These findings are consistent with previous research conclusions (Chen et al., 2024; Liu et al., 2024), further reinforcing the role of exercise in mental recovery following a stroke. Moreover, the subgroup analysis revealed that high compliance with exercise regimens based on ACSM guidelines significantly improved depressive symptoms. This suggests that not only is exercise beneficial, but adherence to standardized exercise recommendations can lead to more substantial improvements in mental outcomes for individuals recovering from stroke.

Several studies have demonstrated that physical exercise has positive effects on non-physical conditions, such as fatigue and depression, in healthy adults of different ages (Chen et al., 2022; Zedlitz et al., 2012; Rhyner and Watts, 2016). Previous researches have reported that exercise, as a non-pharmacological intervention, can effectively alleviate depressive symptoms in stroke patients, which is consistent with the results of this study (Phan et al., 2023; Yi et al., 2024). However, three studies involving elderly populations have demonstrated that these groups exhibit reduced tolerance for high-intensity exercise (Song and Yu, 2019; Kwok et al., 2019; Bouaziz et al., 2019), which may result in increased fatigue and the onset of depressive symptoms. Considering the distinctive physical characteristics of elderly stroke patients (Chang et al., 2015; Mizuta et al., 2020), it is imperative to investigate the effects of varying exercise dosages on their depressive symtom. Exercise dosage encompasses several parameter, including intensity, frequency, and duration. Our subgroup analysis, guided by ACSM recommendations, revealed that exercise was effective in improving depressive symptoms in high compliance group. Several factors may elucidate these findings: First, high-compliance exercise programs may effectively divert patients’ attention through rhythmic and periodic training, thereby mitigating negative emotions such as depression (O’Keefe et al., 2018). Second, the effectiveness of the intervention may depend on the type of exercise. Exercise types encompass aerobic exercise, resistance training, and flexibility training. In this systematic review, only two of the twelve studies in the high compliance group utilized a mixed approach involving aerobic, resistance, and flexibility exercises. However, research by Bemben et al. suggests that composite exercise programs, which integrate various exercise types, may offer additional benefits (Bemben and Bemben, 2011). Therefore, further large-scale and detailed studies are needed to investigate this issue.

This study also performed a subgroup analysis based on the lengths of exercise interventions, which revealed that interventions of varying lengths all contributed to alleviating depressive symptoms in patients, akin to the outcomes observed in non-stroke populations with depression (Li et al., 2024). Although the subgroup with interventions lasting less than 1 month exhibited higher heterogeneity, the effects were most pronounced. As participants progress through the rehabilitation period, an extended duration of exercise enhances cardiovascular adaptability, increases physical resilience, and improves fat oxidation capacity (Jeong et al., 2014). These physiological changes positively contribute to depression improvement (Vetrovsky et al., 2021). Additionally, the adaptation of muscles and joints over time improves exercise efficiency (Bala et al., 2024), reduces injury risk (Lecharte et al., 2020), and enhances the sustainability of the exercise program (Salazar et al., 2019), thereby supporting better psychological health. These benefits not only offer short-term feelings of wellbeing but also foster long-term, stable psychological effects.

Regarding the heterogeneity analysis of the studies, as shown in Figure 4, the meta-analysis revealed a high level of heterogeneity (I^2^ = 78.5%) between studies. The sources of heterogeneity may include differences in sample size, types of exercise interventions, duration, and methods of assessing depression. Firstly, in this study, the difference between the largest and smallest sample sizes was up to 10 times. Studies with smaller sample sizes may lead to inconsistencies in results, increasing the overall heterogeneity. Secondly, different studies employed various types of exercise, frequencies, or durations, which could result in differing treatment effects, thereby contributing to higher clinical heterogeneity. Finally, among the 24 studies included, multiple scales were used to assess the same depressive outcome, which may affect the comparability and integration of the results. Future studies should aim to reduce these discrepancies or adopt more consistent intervention protocols and evaluation criteria.

In conclusion, exercise is beneficial in improving post-stroke depressive symptoms. The results of this study also highlight the significant benefits of stroke patients adhering to the exercise levels recommended by ACSM. These findings suggest that, with appropriate safety measures, high adherence to ACSM guidelines can improve depression in stroke patients. Future research needs to validate our results through large-sample, randomized controlled trials that strictly follow ACSM guidelines, establishing a stronger and more reliable foundation for developing standardized and reproducible exercise interventions.

## 5 Strengths and limitations

This study aimed to determine the optimal exercise regimen for alleviating depressive symptoms in stroke patients, in accordance with ACSM recommendations. The research methodology was in line with the best practices established, incorporating a comprehensive and rigorous search strategy. Additionally, a dual-review process was employed to ensure the accuracy and reliability of the study. The inclusion of only randomized controlled trials further strengthened the quality of the synthesized evidence. However, there are several limitations. First, several of the included studies lacked detailed descriptions of exercise dosage, affecting the precise assessment of ACSM compliance. Second, despite efforts to mitigate bias, uncontrollable factors in the design and execution of interventions may have influenced the results. Moreover, although sensitivity analysis indicated that no single study significantly influenced the overall results, factors such as the severity of participants’ conditions contributed to high heterogeneity. Future research should involve larger sample sizes and multicenter randomized controlled trials to further validate these findings.

## 6 Conclusion

Our study revealed a beneficial effect of exercise on alleviating depression in stroke patients. Our finding indicate that adherence to the exercise recommendations established by ACSM is associated with a more significant improvement in depressive symptoms compared to low adherence or uncertainty regarding these guidelines. This result underscores compliance to ACSM-recommended exercise significantly ameliorate depression levels in stroke patients.

## Data Availability

The original contributions presented in the study are included in the article/Supplementary Material, further inquiries can be directed to the corresponding author.

## References

[B1] Aaron S. E. Gregory C. M. Simpson A. N. (2016). Lower odds of poststroke symptoms of depression when physical activity guidelines met: national health and nutrition examination survey 2011-2012. J. Phys. Activity and Health 13 (8), 903–909. 10.1123/jpah.2015-0446 PMC5289216 27145542

[B2] Aguiar L. T. Nadeau S. Britto R. R. Teixeira-Salmela L. F. Martins J. C. Samora G. A. R. (2020). Effects of aerobic training on physical activity in people with stroke: a randomized controlled trial. NeuroRehabilitation 46 (3), 391–401. 10.3233/NRE-193013 32250336

[B3] Aidar F. J. Gama De Matos D. Jacó De Oliveira R. Carneiro A. L. Cabral B. G. d. A. T. Dantas P. M. S. (2014). Relationship between depression and strength training in survivors of the ischemic stroke. J. Hum. Kinet. 43 (1), 7–15. 10.2478/hukin-2014-0084 25713639 PMC4332168

[B4] Bala S. Vishnu V. Y. Joshi D. (2024). MEFFNet: forecasting myoelectric indices of muscle fatigue in healthy and post-stroke during voluntary and FES-induced dynamic contractions. IEEE Trans. neural Syst. rehabilitation Eng. a Publ. IEEE Eng. Med. Biol. Soc. 32, 2598–2611. 10.1109/TNSRE.2024.3431024 39028608

[B5] Bemben D. A. Bemben M. G. (2011). Dose-response effect of 40 weeks of resistance training on bone mineral density in older adults. Osteoporos. Int. a J. established as result Coop. between Eur. Found. Osteoporos. Natl. Osteoporos. Found. U. S. A. 22 (1), 179–186. 10.1007/s00198-010-1182-9 20195844

[B6] Berretta A. Tzeng Y.-C. Clarkson A. N. (2014). Post-stroke recovery: the role of activity-dependent release of brain-derived neurotrophic factor. Expert Rev. Neurother. 14 (11), 1335–1344. 10.1586/14737175.2014.969242 25319267

[B7] Bouaziz W. Schmitt E. Vogel T. Lefebvre F. Leprêtre P. M. Kaltenbach G. (2019). Effects of a short-term Interval Aerobic Training Programme with active Recovery bouts (IATP-R) on cognitive and mental health, functional performance and quality of life: a randomised controlled trial in sedentary seniors. Int. J. Clin. Pract. 73 (1), e13219. 10.1111/ijcp.13219 29963733

[B8] Chaiyawat P. Kulkantrakorn K. (2012). Randomized controlled trial of home rehabilitation for patients with ischemic stroke: impact upon disability and elderly depression. Psychogeriatrics 12 (3), 193–199. 10.1111/j.1479-8301.2012.00412.x 22994618

[B9] Chang W. H. Shin Y.-I. Lee S.-G. Oh G. J. Lim Y. S. Kim Y. H. (2015). Characteristics of inpatient care and rehabilitation for acute first-ever stroke patients. Yonsei Med. J. 56 (1), 262–270. 10.3349/ymj.2015.56.1.262 25510773 PMC4276765

[B10] Chen P.-J. Chen K.-M. Hsu H.-F. Belcastro F. (2022). Types of exercise and training duration on depressive symptoms among older adults in long-term care facilities. Ageing Res. Rev. 77, 101613. 10.1016/j.arr.2022.101613 35339704

[B11] Chen R. Guo Y. Kuang Y. Zhang Q. (2024). Effects of home-based exercise interventions on post-stroke depression: a systematic review and network meta-analysis. Int. J. Nurs. Stud. 152, 104698. 10.1016/j.ijnurstu.2024.104698 38290424

[B12] Cumming T. Collier J. Thrift A. Bernhardt J. (2008). The effect of very early mobilisation after stroke on psychological well-being. J. Rehabilitation Med. 40 (8), 609–614. 10.2340/16501977-0226 19020693

[B13] Das J. Rajanikant K. (2018). Post stroke depression: the sequelae of cerebral stroke. Neurosci. and Biobehav. Rev. 90, 104–114. 10.1016/j.neubiorev.2018.04.005 29656030

[B14] Deijle I. A. Hemmes R. Boss H. M. de Melker E. C. van den Berg B. T. J. Kwakkel G. (2022). Effect of an exercise intervention on global cognition after transient ischemic attack or minor stroke: the MoveIT randomized controlled trial. BMC Neurol. 22 (1), 289. 10.1186/s12883-022-02805-z 35927622 PMC9351151

[B15] Douven E. Köhler S. Rodriguez M. M. F. Staals J. Verhey F. R. J. Aalten P. (2017). Imaging markers of post-stroke depression and apathy: a systematic review and meta-analysis. Neuropsychol. Rev. 27 (3), 202–219. 10.1007/s11065-017-9356-2 28831649 PMC5613051

[B16] El Husseini N. Goldstein L. B. Peterson E. D. Zhao X. Pan W. Olson D. M. (2012). Depression and antidepressant use after stroke and transient ischemic attack. Stroke 43 (6), 1609–1616. 10.1161/STROKEAHA.111.643130 22461330

[B17] Eng J. J. Reime B. (2014). Exercise for depressive symptoms in stroke patients: a systematic review and meta-analysis. Clin. Rehabil. 28 (8), 731–739. 10.1177/0269215514523631 24535729 PMC4591069

[B18] Faulkner J. McGonigal G. Woolley B. Stoner L. Wong L. Lambrick D. (2015). A randomized controlled trial to assess the psychosocial effects of early exercise engagement in patients diagnosed with transient ischaemic attack and mild, non-disabling stroke. Clin. Rehabil. 29 (8), 783–794. 10.1177/0269215514555729 25352617

[B19] Fichna J. Janecka A. Costentin J. Do Rego J. C. (2007). The endomorphin system and its evolving neurophysiological role. Pharmacol. Rev. 59 (1), 88–123. 10.1124/pr.59.1.3 17329549

[B20] Foley T. E. Fleshner M. (2008). Neuroplasticity of dopamine circuits after exercise: implications for central fatigue. Neuromolecular Med. 10 (2), 67–80. 10.1007/s12017-008-8032-3 18274707

[B21] Garber C. E. Blissmer B. Deschenes M. R. Franklin B. A. Lamonte M. J. Lee I. M. (2011). American College of Sports Medicine position stand. Quantity and quality of exercise for developing and maintaining cardiorespiratory, musculoskeletal, and neuromotor fitness in apparently healthy adults: guidance for prescribing exercise. Med. and Sci. Sports and Exerc. 43 (7), 1334–1359. 10.1249/MSS.0b013e318213fefb 21694556

[B22] Guo X. Zhao P. Zhou X. Wang J. Wang R. (2022). A recommended exercise program appropriate for patients with knee osteoarthritis: a systematic review and meta-analysis. Front. Physiology 13, 934511. 10.3389/fphys.2022.934511 PMC9574341 36262252

[B23] Hackett M. L. Pickles K. (2014). Part I: frequency of depression after stroke: an updated systematic review and meta-analysis of observational studies. Int. J. Stroke 9 (8), 1017–1025. 10.1111/ijs.12357 25117911

[B24] Hadidi N. Treat-Jacobson D. J. Lindquist R. (2009). Poststroke depression and functional outcome: a critical review of literature. Heart and Lung 38 (2), 151–162. 10.1016/j.hrtlng.2008.05.002 19254633

[B25] Heissel A. Heinen D. Brokmeier L. L. Skarabis N. Kangas M. Vancampfort D. (2023). Exercise as medicine for depressive symptoms? A systematic review and meta-analysis with meta-regression. Br. J. Sports Med. 57 (16), 1049–1057. 10.1136/bjsports-2022-106282 36731907 PMC10423472

[B26] Herring M. P. Meyer J. D. (2024). Resistance exercise for anxiety and depression: efficacy and plausible mechanisms. Trends Mol. Med. 30 (3), 204–206. 10.1016/j.molmed.2023.11.016 38296721 PMC11164541

[B27] Higgins J. P. T. Altman D. G. Gøtzsche P. C. Jüni P. Moher D. Oxman A. D. (2011). The cochrane collaboration’s tool for assessing risk of bias in randomised trials. BMJ Clin. Res. ed. 343, d5928. 10.1136/bmj.d5928 PMC3196245 22008217

[B28] Higgins J. P. T. Thomas J. Chandler J. (2024). Cochrane Handbook for systematic reviews of interventions version 6.5. Cochrane. Available at: https://www.training.cochrane.org/handbook.

[B29] Holmgren E. Gosman-Hedström G. Lindström B. Wester P. (2010). What is the benefit of a high-intensive exercise program on health-related quality of life and depression after stroke? A randomized controlled trial. Adv. Physiother. 12 (3), 125–133. 10.3109/14038196.2010.488272 21037954 PMC2956448

[B30] Hong M. S. (2011). The development and effects of an upper extremity exercise program based on patterned sensory enhancement for home-bound stroke patients. J. Korean Acad. Community Health Nurs. 22 (2), 192. 10.12799/jkachn.2011.22.2.192

[B31] Immink M. A. Hillier S. Petkov J. (2014). Randomized controlled trial of yoga for chronic poststroke hemiparesis: motor function, mental health, and quality of life outcomes. Top. STROKE REHABILITATION 21, 256–271. 10.1310/tsr2103-256 24985393

[B32] Jeong Y.-J. Kim W.-C. Kim Y.-S. Choi K. W. Son S. Y. (2014). The relationship between rehabilitation and changes in depression in stroke patients. J. Phys. Ther. Sci. 26 (8), 1263–1266. 10.1589/jpts.26.1263 25202192 PMC4155231

[B33] Jun E. Roh Y. H. Kim M. J. (2013). The effect of music‐movement therapy on physical and psychological states of stroke patients. J. Clin. Nurs. 22 (1–2), 22–31. 10.1111/j.1365-2702.2012.04243.x 22978325

[B34] Koch S. Tiozzo E. Simonetto M. Loewenstein D. Wright C. B. Dong C. (2020). Randomized trial of combined aerobic, resistance, and cognitive training to improve recovery from stroke: feasibility and safety. J. Am. Heart Assoc. 9 (10), e015377. 10.1161/JAHA.119.015377 32394777 PMC7660866

[B35] Kwok J. Y. Y. Kwan J. C. Y. Auyeung M. Mok V. C. T. Lau C. K. Y. Choi K. C. (2019). Effects of mindfulness yoga vs stretching and resistance training exercises on anxiety and depression for people with Parkinson disease: a randomized clinical trial. JAMA neurol. 76 (7), 755–763. 10.1001/jamaneurol.2019.0534 30958514 PMC6583059

[B36] Lai S. Studenski S. Richards L. Perera S. Reker D. Rigler S. (2006). Therapeutic exercise and depressive symptoms after stroke. J. Am. Geriatrics Soc. 54 (2), 240–247. 10.1111/j.1532-5415.2006.00573.x 16460374

[B37] Lanctôt K. L. Lindsay M. P. Smith E. E. Sahlas D. J. Foley N. Gubitz G. (2020). *Canadian stroke best practice recommendations*: mood, cognition and fatigue following stroke, 6th edition update 2019. Int. J. Stroke 15 (6), 668–688. 10.1177/1747493019847334 31221036

[B38] Lecharte T. Gross R. Nordez A. Le Sant G. (2020). Effect of chronic stretching interventions on the mechanical properties of muscles in patients with stroke: a systematic review. Ann. Phys. Rehabilitation Med. 63 (3), 222–229. 10.1016/j.rehab.2019.12.003 31981838

[B39] Levy M. J. F. Boulle F. Steinbusch H. W. van den Hove D. L. A. Kenis G. Lanfumey L. (2018). Neurotrophic factors and neuroplasticity pathways in the pathophysiology and treatment of depression. Psychopharmacology 235 (8), 2195–2220. 10.1007/s00213-018-4950-4 29961124 PMC6061771

[B40] Li W. Liu Y. Deng J. Wang T. (2024). Influence of aerobic exercise on depression in young people: a meta-analysis. BMC psychiatry 24 (1), 571. 10.1186/s12888-024-06013-6 39164715 PMC11337568

[B41] Liang Y. Chan Y. L. Deng M. Chen Y. K. Mok V. Wang D. F. (2018). Enlarged perivascular spaces in the centrum semiovale are associated with poststroke depression: a 3-month prospective study. J. Affect. Disord. 228, 166–172. 10.1016/j.jad.2017.11.080 29253682

[B42] Liu Y. Chen C. Du H. Xue M. Zhu N. (2024). Impact of Baduanjin exercise combined with rational emotive behavior therapy on sleep and mood in patients with poststroke depression: a randomized controlled trial. Medicine 103 (19), e38180. 10.1097/MD.0000000000038180 38728460 PMC11081619

[B43] Loubinoux I. Kronenberg G. Endres M. Schumann-Bard P. Freret T. Filipkowski R. K. (2012). Post-stroke depression: mechanisms, translation and therapy. J. Cell. Mol. Med. 16 (9), 1961–1969. 10.1111/j.1582-4934.2012.01555.x 22348642 PMC3822966

[B44] Marx W. Penninx B. W. J. H. Solmi M. Furukawa T. A. Firth J. Carvalho A. F. (2023). Major depressive disorder. Nat. Rev. Dis. Prim. 9 (1), 44. 10.1038/s41572-023-00454-1 37620370

[B45] Mead G. E. Greig C. A. Cunningham I. Lewis S. J. Dinan S. Saunders D. H. (2007). Stroke: a randomized trial of exercise or relaxation. J. Am. Geriatrics Soc. 55 (6), 892–899. 10.1111/j.1532-5415.2007.01185.x 17537090

[B46] Medeiros G. C. Roy D. Kontos N. Beach S. R. (2020). Post-stroke depression: a 2020 updated review. General Hosp. Psychiatry 66, 70–80. 10.1016/j.genhosppsych.2020.06.011 32717644

[B47] Meneses A. (1998). Physiological, pathophysiological and therapeutic roles of 5-HT systems in learning and memory. Rev. Neurosci. 9 (4), 275–289. 10.1515/REVNEURO.1998.9.4.275 9886142

[B48] Mishchenko V. K. Mishchenko V. M. (2022). Influence of physical rehabilitation on the restoration of psychoemotional and cognitive impairment in patients suffered cerebral ischemic stroke. Acta Balneologica 64 (2), 128–132. 10.36740/ABal202202105

[B49] Mizuta N. Hasui N. Nakatani T. Takamura Y. Fujii S. Tsutsumi M. (2020). Walking characteristics including mild motor paralysis and slow walking speed in post-stroke patients. Sci. Rep. 10 (1), 11819. 10.1038/s41598-020-68905-3 32678273 PMC7366923

[B50] Narendrula A. Ajani K. Lang J. Brinza E. Longenecker C. T. (2023). Psychological distress and health perception in patients with a previous myocardial infarction or stroke: a national cross-sectional study. BMC Cardiovasc. Disord. 23 (1), 430. 10.1186/s12872-023-03422-5 37649045 PMC10468856

[B51] Niu G. Zheng X. Deng B. Yang Q. Du Y. (2024). Effects of exercise dosage on the treatment of fibromyalgia: a meta-analysis of randomised controlled trials. Musculoskelet. Care 22 (3), e1918. 10.1002/msc.1918 39004771

[B52] Niu L. Luo S. S. Xu Y. Wang Z. Luo D. Yang H. (2020). The critical role of the hippocampal NLRP3 inflammasome in social isolation-induced cognitive impairment in male mice. Neurobiol. Learn. Mem. 175, 107301. 10.1016/j.nlm.2020.107301 32882398

[B53] Noetel M. Sanders T. Gallardo-Gómez D. Taylor P. Del Pozo Cruz B. van den Hoek D. (2024). Effect of exercise for depression: systematic review and network meta-analysis of randomised controlled trials. BMJ 384, e075847. 10.1136/bmj-2023-075847 38355154 PMC10870815

[B54] Okaty B. W. Commons K. G. Dymecki S. M. (2019). Embracing diversity in the 5-HT neuronal system. Nat. Rev. Neurosci. 20 (7), 397–424. 10.1038/s41583-019-0151-3 30948838

[B55] O’Keefe J. H. O’Keefe E. L. Lavie C. J. (2018). The goldilocks zone for exercise: not too little, not too much. Mo. Med. 115 (2), 98–105. 30228692 PMC6139866

[B56] Page M. J. McKenzie J. E. Bossuyt P. M. Boutron I. Hoffmann T. C. Mulrow C. D. (2021). The PRISMA 2020 statement: an updated guideline for reporting systematic reviews. BMJ Clin. Res. ed. 372, n71. 10.1136/bmj.n71 PMC8005924 33782057

[B57] Paul S. L. Dewey H. M. Sturm J. W. Macdonell R. A. L. Thrift A. G. (2006). Prevalence of depression and use of antidepressant medication at 5-years poststroke in the north east Melbourne stroke incidence study. Stroke 37 (11), 2854–2855. 10.1161/01.STR.0000244806.05099.52 17008624

[B58] Pearce M. Garcia L. Abbas A. Strain T. Schuch F. B. Golubic R. (2022). Association between physical activity and risk of depression: a systematic review and meta-analysis. JAMA psychiatry 79 (6), 550–559. 10.1001/jamapsychiatry.2022.0609 35416941 PMC9008579

[B59] Phan A. Askim T. Lydersen S. Indredavik B. Wethal T. (2023). Accelerometer-measured physical activity at 3 months as a predictor of symptoms of depression and anxiety 1 year after stroke: a multicentre prospective cohort study in central Norway. J. Rehabilitation Med. 55, jrm12309. 10.2340/jrm.v55.12309 PMC10666064 37970656

[B60] Pontes S. S. de Carvalho A. L. R. Almeida K. de O. Neves M. P. Ribeiro Schindler I. F. S. Alves I. G. N. (2019). Effects of isokinetic muscle strengthening on muscle strength, mobility, and gait in post-stroke patients: a systematic review and meta-analysis. Clin. Rehabil. 33 (3), 381–394. 10.1177/0269215518815220 30484329

[B61] Rhyner K. T. Watts A. (2016). Exercise and depressive symptoms in older adults: a systematic meta-analytic review. J. Aging Phys. Activity 24 (2), 234–246. 10.1123/japa.2015-0146 26372020

[B62] Rosenbaum S. Tiedemann A. Sherrington C. Curtis J. Ward P. B. (2014). Physical activity interventions for people with mental illness: a systematic review and meta-analysis. J. Clin. Psychiatry 75 (9), 964–974. 10.4088/JCP.13r08765 24813261

[B63] Salazar A. P. Pinto C. Ruschel Mossi J. V. Figueiro B. Lukrafka J. L. Pagnussat A. S. (2019). Effectiveness of static stretching positioning on post-stroke upper-limb spasticity and mobility: systematic review with meta-analysis. Ann. Phys. Rehabilitation Med. 62 (4), 274–282. 10.1016/j.rehab.2018.11.004 30582986

[B64] Sims J. Galea M. Taylor N. Dodd K. Jespersen S. Joubert L. (2009). Regenerate: assessing the feasibility of a strength‐training program to enhance the physical and mental health of chronic post stroke patients with depression. Int. J. Geriatric Psychiatry 24 (1), 76–83. 10.1002/gps.2082 18613281

[B65] Skolarus L. E. Piette J. D. Pfeiffer P. N. Williams L. S. Mackey J. Hughes R. (2017). Interactive voice response—an innovative approach to post-stroke depression self-management support. Transl. Stroke Res. 8 (1), 77–82. 10.1007/s12975-016-0481-7 27394917 PMC5507192

[B66] Smith P. S. Thompson M. (2008). Treadmill training post stroke: are there any secondary benefits? A pilot study. Clin. Rehabil. 22 (10–11), 997–1002. 10.1177/0269215508088988 18955431

[B67] Song D. Yu D. S. F. (2019). Effects of a moderate-intensity aerobic exercise programme on the cognitive function and quality of life of community-dwelling elderly people with mild cognitive impairment: a randomised controlled trial. Int. J. Nurs. Stud. 93, 97–105. 10.1016/j.ijnurstu.2019.02.019 30901716

[B68] Song Y.-Y. Sun W.-J. Wang C. Tian Y. M. Liu H. Jiang Y. (2023). Effects of multicomponent exercise on quality of life, depression and anxiety among stroke survivors: a systematic review and meta-analysis. J. Clin. Nurs. 32 (21–22), 7677–7690. 10.1111/jocn.16853 37727891

[B69] Steen Krawcyk R. Vinther A. Petersen N. C. Faber J. Iversen H. K. Christensen T. (2019). Effect of home-based high-intensity interval training in patients with lacunar stroke: a randomized controlled trial. Front. Neurology 10, 664. 10.3389/fneur.2019.00664 PMC6611174 31316451

[B70] Sun P. Zhang S. Jiang L. Ma Z. Yao C. Zhu Q. (2022). Yijinjing Qigong intervention shows strong evidence on clinical effectiveness and electroencephalography signal features for early poststroke depression: a randomized, controlled trial. Front. Aging Neurosci. 14, 956316. 10.3389/fnagi.2022.956316 36034130 PMC9400391

[B71] Tang X.-Q. Liao R.-Y. Zheng L.-J. Yang L. L. Ma Z. L. Yi C. (2022). Aerobic exercise reverses the NF-κB/NLRP3 inflammasome/5-HT pathway by upregulating irisin to alleviate post-stroke depression. Ann. Transl. Med. 10 (24), 1350. 10.21037/atm-22-5443 36660693 PMC9843332

[B72] Taylor-Piliae R. E. Hoke T. M. Hepworth J. T. Latt L. D. Najafi B. Coull B. M. (2014). Effect of tai Chi on physical function, fall rates and quality of life among older stroke survivors. Archives Phys. Med. Rehabilitation 95 (5), 816–824. 10.1016/j.apmr.2014.01.001 24440643

[B73] Tseng C. Chen C. C. Wu S. Lin L. C. (2007). Effects of a range‐of‐motion exercise programme. J. Adv. Nurs. 57 (2), 181–191. 10.1111/j.1365-2648.2006.04078.x 17214754

[B74] Van De Port I. G. L. Wevers L. E. G. Lindeman E. Kwakkel G. (2012). Effects of circuit training as alternative to usual physiotherapy after stroke: randomised controlled trial. BMJ 344 (may10 1), e2672. 10.1136/bmj.e2672 22577186 PMC3349299

[B75] Vetrovsky T. Fortova T. Conesa-Ros E. Steffl M. Heczkova J. Belohlavek J. (2021). Increased cardiopulmonary fitness is associated with a greater reduction in depression among people who underwent bariatric surgery. Int. J. Environ. Res. Public Health 18 (5), 2508. 10.3390/ijerph18052508 33802552 PMC7967516

[B76] Vloothuis J. D. M. Mulder M. Nijland R. H. M. Goedhart Q. S. Konijnenbelt M. Mulder H. (2019). Caregiver-mediated exercises with e-health support for early supported discharge after stroke (CARE4STROKE): a randomized controlled trial. PLoS One 14, e0214241. 10.1371/journal.pone.0214241 30958833 PMC6453481

[B77] Wen Q. Mao X.-R. Wen J. Yang X. J. Chen J. Han H. K. (2024). Impact of exercise dosages based on american college of sports medicine recommendations on lipid metabolism in patients after PCI: a systematic review and meta-analysis of randomized controlled trials. Lipids Health Dis. 23 (1), 226. 10.1186/s12944-024-02210-0 39049120 PMC11267757

[B78] Woodford J. Farrand P. Watkins E. R. Llewellyn D. J. (2018). “I don’t believe in leading a life of my own, I lead his life”: a qualitative investigation of difficulties experienced by informal caregivers of stroke survivors experiencing depressive and anxious symptoms. Clin. Gerontol. 41 (4), 293–307. 10.1080/07317115.2017.1363104 29185911

[B79] Yi Y. Zhao W. Lv S. Zhang G. Rong Y. Wang X. (2024). Effectiveness of non-pharmacological therapies for treating post-stroke depression: a systematic review and network meta-analysis. General Hosp. Psychiatry 90, 99–107. 10.1016/j.genhosppsych.2024.07.011 39084147

[B80] Zedlitz A. M. E. E. Rietveld T. C. M. Geurts A. C. Fasotti L. (2012). Cognitive and graded activity training can alleviate persistent fatigue after stroke: a randomized, controlled trial. Stroke 43 (4), 1046–1051. 10.1161/STROKEAHA.111.632117 22308241

[B81] Zhang L. Zhao J. Quan S. Liu Y. h. Shi X. h. Li Z. g. (2018). Effect of acupuncture plus tai ji quan on the recovery of neurological function and depression state in post-stroke depression patients. J. Acupunct. Tuina Sci. 16 (2), 96–103. 10.1007/s11726-018-1031-5

[B82] Zhao J. Chau J. P. C. Chan A. W. K. Meng Q. Choi K. C. Xiang X. (2020). Tailored sitting tai Chi program for subacute stroke survivors: a randomized controlled trial. Stroke Vasc. Interv. Neurol. 53, 2192–2203. 10.1161/STROKEAHA.121.036578 35387494

