# Supplementary material for: The effects of exercise interventions on depressive symptoms in stroke patients: a systematic review and meta-analysis
Source: Front Physiol. 2025 Mar 17;16:1492221. doi: 10.3389/fphys.2025.1492221 (PMC11955706; doi:10.3389/fphys.2025.1492221)
Supplement: Supplementary file 1 [file Table1.doc]

**Supplementary Table1. Search strategy on PubMed.**

| #1 | "Stroke"[MeSH] |
| --- | --- |
| #2 | (((((((((((((((((((((((((((((Stroke[Title/Abstract]) OR (Strokes[Title/Abstract])) OR (Cerebrovascular Accident[Title/Abstract])) OR (Cerebrovascular Accidents[Title/Abstract])) OR (Cerebral Stroke[Title/Abstract])) OR (Cerebral Strokes[Title/Abstract])) OR (Stroke, Cerebral[Title/Abstract])) OR (Strokes, Cerebral[Title/Abstract])) OR (Cerebrovascular Apoplexy[Title/Abstract])) OR (Apoplexy, Cerebrovascular[Title/Abstract])) OR (Vascular Accident, Brain[Title/Abstract])) OR (Brain Vascular Accident[Title/Abstract])) OR (Brain Vascular Accidents[Title/Abstract])) OR (Vascular Accidents, Brain[Title/Abstract])) OR (Cerebrovascular Stroke[Title/Abstract])) OR (Cerebrovascular Strokes[Title/Abstract])) OR (Stroke, Cerebrovascular[Title/Abstract])) OR (Strokes, Cerebrovascular[Title/Abstract])) OR (Apoplexy[Title/Abstract])) OR (CVA (Cerebrovascular Accident[Title/Abstract]))) OR (CVAs (Cerebrovascular Accident[Title/Abstract]))) OR (Stroke, Acute[Title/Abstract])) OR (Acute Stroke[Title/Abstract])) OR (Acute Strokes[Title/Abstract])) OR (Strokes, Acute[Title/Abstract])) OR (Cerebrovascular Accident, Acute[Title/Abstract])) OR (Acute Cerebrovascular Accident[Title/Abstract])) OR (Acute Cerebrovascular Accidents[Title/Abstract])) OR (Cerebrovascular Accidents, Acute[Title/Abstract])) |
| #3 | #1 OR #2 |
| #4 | "Depression"[MeSH] |
| #5 | (((((((((((((((((((((((((((((((((Depressive Disorder[MeSH Terms])) OR (Depression[Title/Abstract])) OR (Depressive Disorder[Title/Abstract])) OR (Depressive Symptoms[Title/Abstract])) OR (Depressive Symptom[Title/Abstract])) OR (Symptom, Depressive[Title/Abstract])) OR (Emotional Depression[Title/Abstract])) OR (Depression, Emotional[Title/Abstract])) OR (Depressive Disorders[Title/Abstract])) OR (Disorder, Depressive[Title/Abstract])) OR (Disorders, Depressive[Title/Abstract])) OR (Neurosis, Depressive[Title/Abstract])) OR (Depressive Neuroses[Title/Abstract])) OR (Depressive Neurosis[Title/Abstract])) OR (Neuroses, Depressive[Title/Abstract])) OR (Depression, Endogenous[Title/Abstract])) OR (Depressions, Endogenous[Title/Abstract])) OR (Endogenous Depression[Title/Abstract])) OR (Endogenous Depressions[Title/Abstract])) OR (Melancholia[Title/Abstract])) OR (Melancholias[Title/Abstract])) OR (Unipolar Depression[Title/Abstract])) OR (Depression, Unipolar[Title/Abstract])) OR (Depressions, Unipolar[Title/Abstract])) OR (Unipolar Depressions[Title/Abstract])) OR (Depressive Syndrome[Title/Abstract])) OR (Depressive Syndromes[Title/Abstract])) OR (Syndrome, Depressive[Title/Abstract])) OR (Syndromes, Depressive[Title/Abstract])) OR (Depression, Neurotic[Title/Abstract])) OR (Depressions, Neurotic[Title/Abstract])) OR (Neurotic Depression[Title/Abstract])) OR (Neurotic Depressions[Title/Abstract]) |
| #6 | #4 OR #5 |
| #7 | "Exercise"[MeSH] |
| #8 | ((((((((((((((((((((((((((((((Exercises[Title/Abstract])) OR (Exercise[Title/Abstract])) OR (Exercise, Physical[Title/Abstract])) OR (Exercises, Physical[Title/Abstract])) OR (Physical Exercise[Title/Abstract])) OR (Physical Exercises[Title/Abstract])) OR (Physical Activity[Title/Abstract])) OR (Activities, Physical[Title/Abstract])) OR (Activity, Physical[Title/Abstract])) OR (Physical Activities[Title/Abstract])) OR (Exercise, Aerobic[Title/Abstract])) OR (Aerobic Exercise[Title/Abstract])) OR (Aerobic Exercises[Title/Abstract])) OR (Exercises, Aerobic[Title/Abstract])) OR (Exercise, Isometric[Title/Abstract])) OR (Exercises, Isometric[Title/Abstract])) OR (Isometric Exercises[Title/Abstract])) OR (Isometric Exercise[Title/Abstract])) OR (Acute Exercise[Title/Abstract])) OR (Acute Exercises[Title/Abstract])) OR (Exercise, Acute[Title/Abstract])) OR (Exercises, Acute[Title/Abstract])) OR (Exercise Training[Title/Abstract])) OR (Exercise Trainings[Title/Abstract])) OR (Training, Exercise[Title/Abstract])) OR (Trainings, Exercise[Title/Abstract])) OR (flexibility[Title/Abstract])) OR (neuro-motor skills training[Title/Abstract])) OR (Tai Chi[Title/Abstract])) OR (yoga[Title/Abstract]) |
| #9 | #7 OR #8 |
| #10 | #3 AND #6 AND #9 |

**Supplementary Table2. Search strategy on EMBASE.**

| #1 | depression:ab,ti OR 'depressive disorder':ab,ti OR 'depressive symptoms':ab,ti OR 'depressive symptom':ab,ti OR 'symptom, depressive':ab,ti OR 'emotional depression':ab,ti OR 'depression, emotional':ab,ti OR 'depressive disorders':ab,ti OR 'disorder, depressive':ab,ti OR 'disorders, depressive':ab,ti OR 'neurosis, depressive':ab,ti OR 'depressive neuroses':ab,ti OR 'depressive neurosis':ab,ti OR 'neuroses, depressive':ab,ti OR 'depression, endogenous':ab,ti OR 'depressions, endogenous':ab,ti OR 'endogenous depression':ab,ti OR 'endogenous depressions':ab,ti OR melancholia:ab,ti OR melancholias:ab,ti OR 'unipolar depression':ab,ti OR 'depression, unipolar':ab,ti OR 'depressions, unipolar':ab,ti OR 'unipolar depressions':ab,ti OR 'depressive syndrome':ab,ti OR 'depressive syndromes':ab,ti OR 'syndrome, depressive':ab,ti OR 'syndromes, depressive':ab,ti OR 'depression, neurotic':ab,ti OR 'depressions, neurotic':ab,ti OR 'neurotic depression':ab,ti OR 'neurotic depressions':ab,ti |
| --- | --- |
| #2 | stroke:ab,ti OR strokes:ab,ti OR 'cerebrovascular accident':ab,ti OR 'cerebrovascular accidents':ab,ti OR 'cerebral stroke':ab,ti OR 'cerebral strokes':ab,ti OR 'stroke, cerebral':ab,ti OR 'strokes, cerebral':ab,ti OR 'cerebrovascular apoplexy':ab,ti OR 'apoplexy, cerebrovascular':ab,ti OR 'vascular accident, brain':ab,ti OR 'brain vascular accident':ab,ti OR 'brain vascular accidents':ab,ti OR 'vascular accidents, brain':ab,ti OR 'cerebrovascular stroke':ab,ti OR 'cerebrovascular strokes':ab,ti OR 'stroke, cerebrovascular':ab,ti OR 'strokes, cerebrovascular':ab,ti OR apoplexy:ab,ti OR (cva:ab,ti AND 'cerebrovascular accident':ab,ti) OR (cvas:ab,ti AND 'cerebrovascular accident':ab,ti) OR 'stroke, acute':ab,ti OR 'acute stroke':ab,ti OR 'acute strokes':ab,ti OR 'strokes, acute':ab,ti OR 'cerebrovascular accident, acute':ab,ti OR 'acute cerebrovascular accident':ab,ti OR 'acute cerebrovascular accidents':ab,ti OR 'cerebrovascular accidents, acute':ab,ti |
| #3 | exercise:ab,ti OR exercises:ab,ti OR 'exercise, physical':ab,ti OR 'exercises, physical':ab,ti OR 'physical exercise':ab,ti OR 'physical exercises':ab,ti OR 'physical activity':ab,ti OR 'activities, physical':ab,ti OR 'activity, physical':ab,ti OR 'physical activities':ab,ti OR 'exercise, aerobic':ab,ti OR 'aerobic exercise':ab,ti OR 'aerobic exercises':ab,ti OR 'exercises, aerobic':ab,ti OR 'exercise, isometric':ab,ti OR 'exercises, isometric':ab,ti OR 'isometric exercises':ab,ti OR 'isometric exercise':ab,ti OR 'acute exercise':ab,ti OR 'acute exercises':ab,ti OR 'exercise, acute':ab,ti OR 'exercises, acute':ab,ti OR 'exercise training':ab,ti OR 'exercise trainings':ab,ti OR 'training, exercise':ab,ti OR 'trainings, exercise':ab,ti OR flexibility:ab,ti OR 'neuro-motor skills training':ab,ti OR 'tai chi':ab,ti OR yoga:ab,ti |
| #4 | #1 AND #2 AND #3 |

**Supplementary Table3. Search strategy on Web of Science.**

| #1 | (((((((((((((((((((((((((((((TS=(Stroke)) OR TS=(Strokes)) OR TS=(Cerebrovascular Accident)) OR TS=(Cerebrovascular Accidents)) OR TS=(Cerebral Stroke)) OR TS=(Cerebral Strokes)) OR ((((((((((((((((((((((((((((TS=(Stroke)) OR TS=(Strokes)) OR TS=(Cerebrovascular Accident)) OR TS=(Cerebrovascular Accidents)) OR TS=(Cerebral Stroke)) OR TS=(Cerebral Strokes)) OR TS=(Stroke, Cerebral)) OR TS=(Strokes, Cerebral)) OR TS=(Cerebrovascular Apoplexy)) OR TS=(Apoplexy, Cerebrovascular)) OR TS=(Vascular Accident, Brain)) OR TS=(Brain Vascular Accident)) OR TS=(Brain Vascular Accidents)) OR TS=(Vascular Accidents, Brain)) OR TS=(Cerebrovascular Stroke)) OR TS=(Cerebrovascular Strokes)) OR TS=(Stroke, Cerebrovascular)) OR TS=(Strokes, Cerebrovascular)) OR TS=(Apoplexy)) OR TS=(CVA (Cerebrovascular Accident))) OR TS=(CVAs (Cerebrovascular Accident))) OR TS=(Stroke, Acute)) OR TS=(Acute Stroke)) OR TS=(Acute Strokes)) OR TS=(Strokes, Acute)) OR TS=(Cerebrovascular Accident, Acute)) OR TS=(Acute Cerebrovascular Accident)) OR TS=(Acute Cerebrovascular Accidents)) OR TS=(Cerebrovascular Accidents, Acute) and Preprint Citation Index (Exclude – Database) |
| --- | --- |
| #2 | (((((((((((((((((((((((((((((TS=(Exercise)) OR TS=(Exercises)) OR TS=(Exercise, Physical)) OR TS=(Exercises, Physical)) OR TS=(Physical Exercise)) OR TS=(Physical Exercises)) OR TS=(Physical Activity)) OR TS=(Activities, Physical)) OR TS=(Activity, Physical)) OR TS=(Physical Activities)) OR TS=(Exercise, Aerobic)) OR TS=(Aerobic Exercise)) OR TS=(Aerobic Exercises)) OR TS=(Exercises, Aerobic)) OR TS=(Exercise, Isometric)) OR TS=(Exercises, Isometric)) OR TS=(Isometric Exercises)) OR TS=(Isometric Exercise)) OR TS=(Acute Exercise)) OR TS=(Acute Exercises)) OR TS=(Exercise, Acute)) OR TS=(Exercises, Acute)) OR TS=(Exercise Training)) OR TS=(Exercise Trainings)) OR TS=(Training, Exercise)) OR TS=(Trainings, Exercise)) OR TS=(flexibility)) OR TS=(neuro-motor skills training)) OR TS=(Tai Chi)) OR TS=(yoga) and Preprint Citation Index (Exclude – Database) |
| #3 | (((((((((((((((((((((((((((((((TS=(Depression)) OR TS=(Depressive Disorder)) OR TS=(Depressive Symptoms)) OR TS=(Depressive Symptom)) OR TS=(Symptom, Depressive)) OR TS=(Emotional Depression)) OR TS=(Depression, Emotional)) OR TS=(Depressive Disorders)) OR TS=(Disorder, Depressive)) OR TS=(Disorders, Depressive)) OR TS=(Neurosis, Depressive)) OR TS=(Depressive Neuroses)) OR TS=(Depressive Neurosis)) OR TS=(Neuroses, Depressive)) OR TS=(Depression, Endogenous)) OR TS=(Depressions, Endogenous)) OR TS=(Endogenous Depression)) OR TS=(Endogenous Depressions)) OR TS=(Melancholia)) OR TS=(Melancholias)) OR TS=(Unipolar Depression)) OR TS=(Depression, Unipolar)) OR TS=(Depressions, Unipolar)) OR TS=(Unipolar Depressions)) OR TS=(Depressive Syndrome)) OR TS=(Depressive Syndromes)) OR TS=(Syndrome, Depressive)) OR TS=(Syndromes, Depressive)) OR TS=(Depression, Neurotic)) OR TS=(Depressions, Neurotic)) OR TS=(Neurotic Depression)) OR TS=(Neurotic Depressions) and Preprint Citation Index (Exclude – Database) |
| #4 | #1 AND #2 AND #3 |

**Supplementary Table4. Search strategy on CENTRAL.**

| #1 | (depression): ti,ab,kw OR (depressive disorder): ti,ab,kw OR (depressive symptoms): ti,ab,kw OR (depressive symptom): ti,ab,kw OR (symptom, depressive): ti,ab,kw OR (emotional depression): ti,ab,kw OR (depression, emotional): ti,ab,kw OR (depressive disorders): ti,ab,kw OR (disorder, depressive): ti,ab,kw OR (disorders, depressive): ti,ab,kw OR (neurosis, depressive): ti,ab,kw OR (depressive neuroses): ti,ab,kw OR (depressive neurosis): ti,ab,kw OR (neuroses, depressive): ti,ab,kw OR (depression, endogenous): ti,ab,kw OR (depressions, endogenous): ti,ab,kw OR (endogenous depression): ti,ab,kw OR (endogenous depressions): ti,ab,kw OR (melancholia): ti,ab,kw OR (melancholias): ti,ab,kw OR (unipolar depression): ti,ab,kw OR (depression, unipolar): ti,ab,kw OR (depressions, unipolar): ti,ab,kw OR (unipolar depressions): ti,ab,kw OR (depressive syndrome): ti,ab,kw OR (depressive syndromes): ti,ab,kw OR (syndrome, depressive): ti,ab,kw OR (syndromes, depressive): ti,ab,kw OR (depression, neurotic): ti,ab,kw OR (depressions, neurotic): ti,ab,kw OR (neurotic depression): ti,ab,kw OR (neurotic depressions): ti,ab,kw |
| --- | --- |
| #2 | (stroke): ti,ab,kw OR (strokes): ti,ab,kw OR (cerebrovascular accident): ti,ab,kw OR (cerebrovascular accidents): ti,ab,kw OR (cerebral stroke): ti,ab,kw OR (cerebral strokes): ti,ab,kw OR (stroke, cerebral): ti,ab,kw OR (strokes, cerebral): ti,ab,kw OR (cerebrovascular apoplexy): ti,ab,kw OR (apoplexy, cerebrovascular): ti,ab,kw OR (vascular accident, brain): ti,ab,kw OR (brain vascular accident): ti,ab,kw OR (brain vascular accidents): ti,ab,kw OR (vascular accidents, brain): ti,ab,kw OR (cerebrovascular stroke): ti,ab,kw OR (cerebrovascular strokes): ti,ab,kw OR (stroke, cerebrovascular): ti,ab,kw OR (strokes, cerebrovascular): ti,ab,kw OR (apoplexy): ti,ab,kw OR ((cva): ti,ab,kw AND (cerebrovascular accident): ti,ab,kw)) OR ((cvas): ti,ab,kw AND (cerebrovascular accident): ti,ab,kw)) OR (stroke, acute): ti,ab,kw OR (acute stroke): ti,ab,kw OR (acute strokes): ti,ab,kw OR (strokes, acute): ti,ab,kw OR (cerebrovascular accident, acute): ti,ab,kw OR (acute cerebrovascular accident): ti,ab,kw OR (acute cerebrovascular accidents): ti,ab,kw OR (cerebrovascular accidents, acute): ti,ab,kw |
| #3 | (exercise): ti,ab,kw OR (exercises): ti,ab,kw OR (exercise, physical): ti,ab,kw OR (exercises, physical): ti,ab,kw OR (physical exercise): ti,ab,kw OR (physical exercises): ti,ab,kw OR (physical activity): ti,ab,kw OR (activities, physical): ti,ab,kw OR (activity, physical): ti,ab,kw OR (physical activities): ti,ab,kw OR (exercise, aerobic): ti,ab,kw OR (aerobic exercise): ti,ab,kw OR (aerobic exercises): ti,ab,kw OR (exercises, aerobic): ti,ab,kw OR (exercise, isometric): ti,ab,kw OR (exercises, isometric): ti,ab,kw OR (isometric exercises): ti,ab,kw OR (isometric exercise): ti,ab,kw OR (acute exercise): ti,ab,kw OR (acute exercises): ti,ab,kw OR (exercise, acute): ti,ab,kw OR (exercises, acute): ti,ab,kw OR (exercise training): ti,ab,kw OR (exercise trainings): ti,ab,kw OR (training, exercise): ti,ab,kw OR (trainings, exercise): ti,ab,kw OR (flexibility): ti,ab,kw OR (neuro-motor skills training): ti,ab,kw OR (tai chi): ti,ab,kw OR (yoga): ti,ab,kw |
| #4 | #1 AND #2 AND #3 |
